# Popliteal Artery Injury Risk in Total Knee Arthroplasty Related to Anatomic Variations: A Scoping Review

**DOI:** 10.1016/j.artd.2026.101975

**Published:** 2026-02-25

**Authors:** Hiren Parekh, Bohdanna Zazulak, Adam V. Daniel, Kinjal Vasavada, Elie Mansour, Michael Medvecky

**Affiliations:** a Yale College, Yale University, New Haven, CT, USA; b Department of Orthopaedic Surgery, Yale School of Medicine, New Haven, CT USA; c Department of Orthopedic Surgery, Orlando Health Jewett Orthopedic Institute, Orlando, FL, USA

**Keywords:** Popliteal artery, Anterior tibial artery, Anatomical variation, Vascular injury, Total knee arthroplasty, Orthopaedic surgery complications

## Abstract

**Background:**

Popliteal artery (PA) injury during total knee arthroplasty (TKA) is rare but can lead to devastating complications, including PA repair or bypass, limb ischemia, compartment syndrome, fasciotomy, amputation, and death. Anatomical variations, such as high PA bifurcation or aberrant anterior tibial artery (ATA), may elevate this risk. Awareness of these variants during preoperative planning may reduce the likelihood of vascular injury and postoperative complications. This study aimed to assess the prevalence of PA anatomical variants, their relevance to TKA, and associated vascular complications. We hypothesized that aberrant arterial anatomy increases complication risk.

**Methods:**

A systematic literature search was conducted in EMBASE, MEDLINE, and Scopus in February 2025 using terms related to “aberrant popliteal artery,” “anterior tibial artery anomaly,” and “vascular complications in knee surgery.” Studies were screened via Covidence. Inclusion criteria included the following: studies evaluating PA variation, prevalence, or TKA-associated vascular complications. Exclusion criteria included the following: non-English, abstracts, reviews, or unrelated studies. Extracted data included anatomical findings, imaging, and surgical outcomes. This study is compliant with Preferred Reporting Items for Systematic reviews and Meta-Analyses extension for Scoping Reviews and Level of Evidence IV.

**Results:**

Of 5093 articles, 11 met inclusion criteria. Four case reports described vascular injuries during TKA in patients with aberrant PA anatomy (mean age 63 ± 18.2; 75% women). Imaging studies reported aberrant ATA prevalence ranging from 0.4% to 6%; one magnetic resonance imaging (MRI) study found a 3.2% prevalence in 280 knees. Proximity of the ATA to key landmarks increased injury risk, especially in women. Two cadaveric studies and 1 cohort study identified high PA bifurcations and variant ATA origins as risk factors.

**Conclusions:**

PA anatomical variation may be an underrecognized anomaly and be a risk factor for vascular injury during TKA. Awareness of the anatomical variation, careful review of pre-existing MRIs, and selective vascular imaging may improve surgical planning and help mitigate complications.

## Introduction

Iatrogenic injury to the popliteal artery (PA) is rare but can result in potentially disastrous complications [[1], [2], [3], [4], [5], [6], [7], [8], [9], [10], [11]]. These include the need for arterial repair or bypass, limb ischemia, emergent reoperation, compartment syndrome requiring fasciotomy, contralateral vein graft harvest, and amputation [[1], [2], [3], [4], [5], [6], [7], [8], [9], [10], [11]]. Given the proximity of the PA to the posterior knee capsule, even minor deviations in surgical technique or anatomical variations can increase the risk of vascular injury [1,12,13]. Anomalies such as an aberrant anterior tibial artery (ATA) or high division of the PA can bring the vasculature dangerously close to the surgical field. This increases the risk of injury during dissection, retractor placement, or saw cuts, highlighting the importance of thorough preoperative imaging and vascular assessment [1,[14], [15], [16], [17], [18]]. The PA gives rise to several genicular branches that form a dense vascular network around the knee joint, which may also be susceptible to injury during surgical exposure or retraction [17].

**Figure 1:**
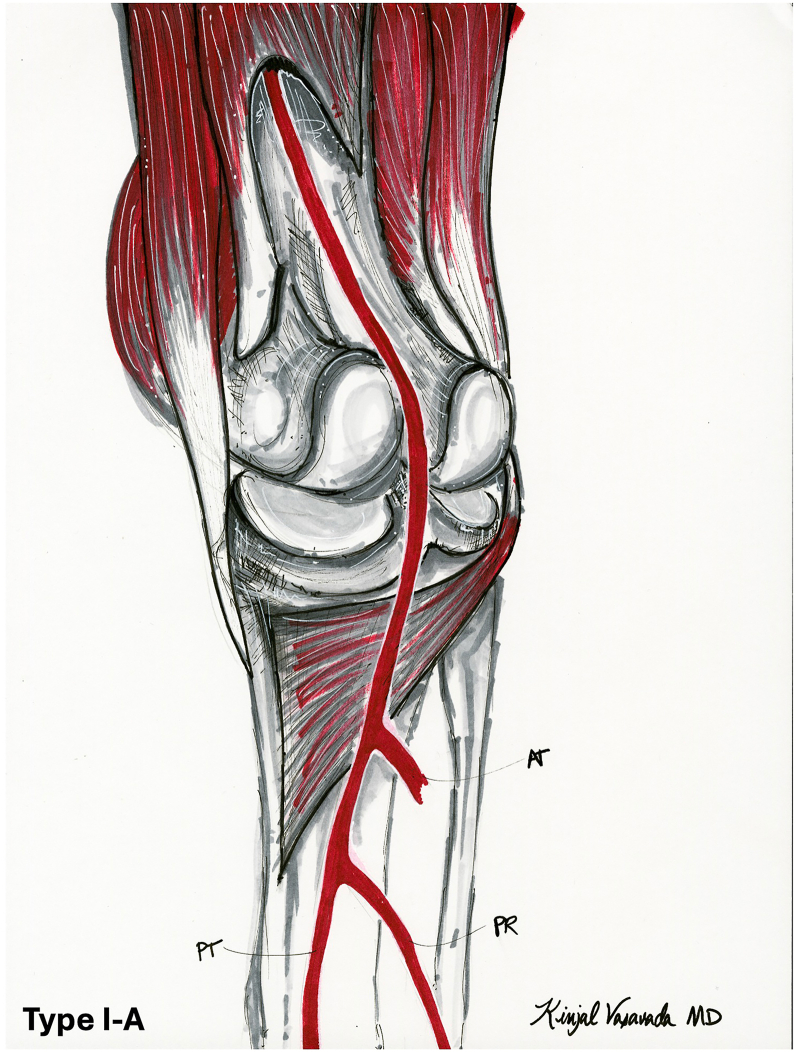
Normal popliteal artery anatomy (adapted from type I-A in Kim et al 1989). The “normal” vascular anatomy of the popliteal artery has been described as a bifurcation below the knee joint level in which the anterior tibial artery arises first, followed by the tibioperoneal trunk, which subsequently divides into the peroneal and posterior tibial arteries. AT, anterior tibial artery; PR, peroneal artery.

One of the most frequently cited studies on “normal” PA branching was conducted by Kim et al. In a femoral angiogram series of 1000 extremities, they found that the ATA was typically the first branch, followed by the tibioperoneal trunk, which then bifurcated into the peroneal and posterior tibial arteries (Fig. 1) [17].

**Figure 2:**
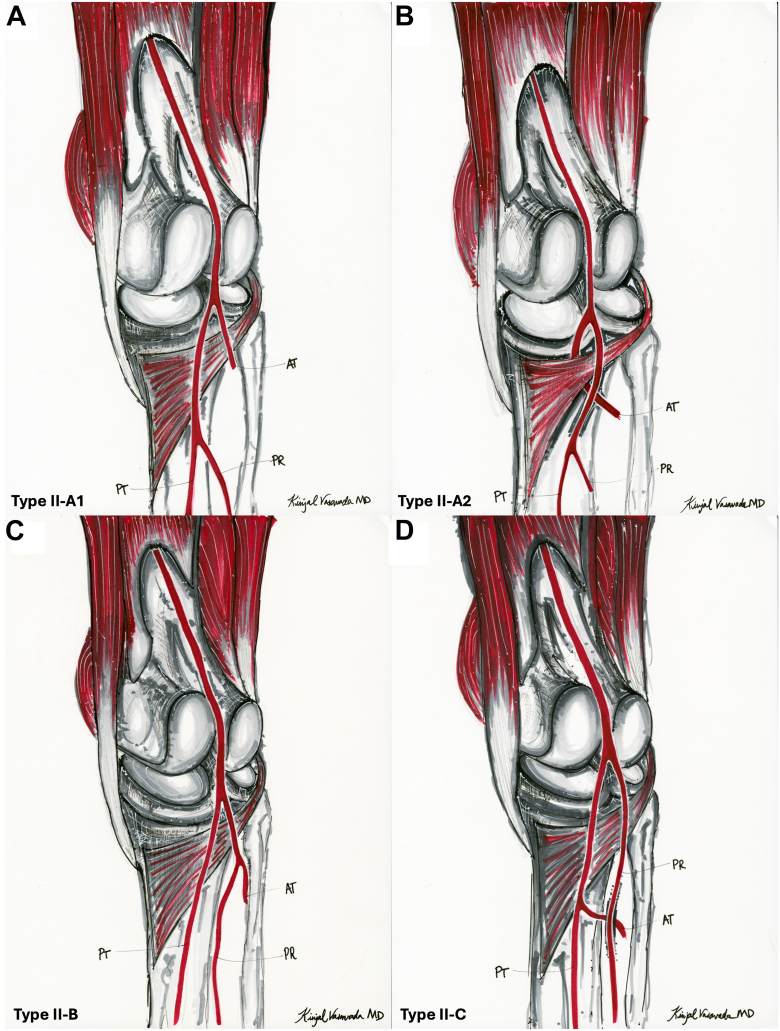
High-division popliteal artery branching variants (adapted from Kim et al 1989). (a) Type II-A1: The anterior tibial artery arises above the knee joint and courses straight distally in its proximal segment. (b) Type II-A2: The anterior tibial artery arises above the knee joint and takes a medial looping course, likely due to its passage anterior to the popliteus muscle. (c) Type II-B: PT is the first branch and originates above the knee joint. (d) Type II-C: PR is the first branch of the PA and originates above the knee joint line. AT, anterior tibial artery; PR, peroneal artery.

**Figure 3:**
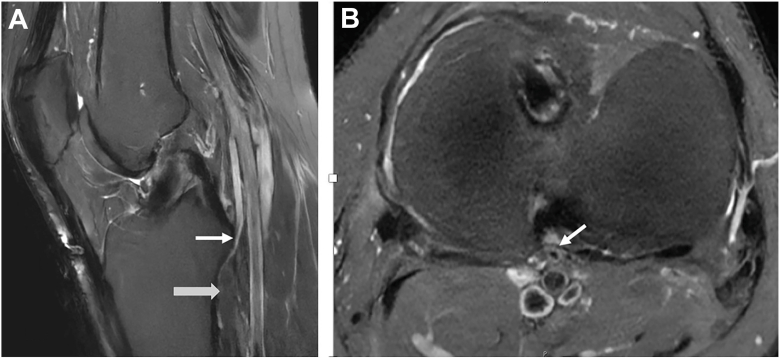
MRI example of Kim et al. 1989 type II-A2. (a) Sagittal T2-weighted image showing anterior tibial artery (white arrow) coursing anterior to the popliteus muscle (gray arrow). (b) Axial T2-weighted image showing the aberrant anterior tibial artery (white arrow) immediately adjacent to the distal-posterior aspect of the posterior cruciate ligament, before coursing along the posterior tibial cortex.

However, type II in Kim et al. classification is a form of aberrant vascular anatomy. It includes 4 variants in which the ATA arises at or above the knee joint line (Fig. 2). High division of the PA occurs more proximally, altering the course of the ATA and placing it at greater risk during total knee arthroplasty (TKA) [1,18]. For example, in Kim type IIA-2 (Fig. 3a, b), the ATA courses anterior to the popliteus muscle and becomes fixed against the posterior tibial (PT) cortex, making it particularly vulnerable to iatrogenic injury during bone cuts of the proximal tibia and posterior femoral condyles, soft tissue releases, and hyperextension when manipulating the knee prior to final implant positioning [1,19,20].

Although the prevalence of PA anatomical variation is low, it remains clinically relevant for TKA outcomes. Klecker et al. identified a 2.1% prevalence of aberrant ATA anatomy on magnetic resonance imaging (MRI), while Marin-Concha et al. reported 2.9% in their cohort [1,14]. Advanced imaging modalities such as MRI, arteriography, and ultrasound have been suggested for detecting these variants [1]. However, the American Academy of Orthopaedic Surgeons 2022 Clinical Practice Guidelines recommend routine preoperative weight-bearing X-rays as sufficient for most TKA cases, advising against routine MRI or computed tomography (CT) due to cost and lack of proven benefit [[21], [22], [23], [24], [25]]. Advanced imaging may be appropriate in select patients with prior hardware or some robotic-assisted surgery platforms, or vascular risk factors such as atherosclerosis, malignancy, or significant weight loss [6,9,11,21,22,[26], [27], [28], [29]]. For assessment of vascular anatomy variation, any distant MRI of the ipsilateral knee, if a prior imaging workup had been performed, would also be of value for preoperative planning.

Arterial injuries during TKA, such as transection, laceration, thrombus, embolus, and pseudoaneurysm, can have devastating outcomes [[30], [31], [32], [33], [34]]. Gosslau et al. reported 16 vascular injuries after knee surgery, including 11 during primary TKAs and 2 during revision [4]. Collier et al. described 24 cases of postoperative ischemia within 5 days of TKA, resulting in 18 amputations and 6 cases of severe dysfunction [35]. Neither study identified technical error or vascular anomaly as the cause. These injuries may be underreported and have been linked to increased malpractice risk due to delayed diagnosis and treatment [35].

With TKA volumes rising, recognizing PA anatomical variations is critical for surgical planning and minimizing risk of complications [19,20,23,24,36]. The PA and its branches’ proximity to the tibial cortex varies among patients and their anatomy, with women, in general, being at higher risk [16]. Hamahashi et al. have demonstrated sex-based anatomic differences in PA position, with the distance between the PT cortex and the anterior border of the PA being smaller in women than in men, particularly at the joint level and distal zones [37]. Although the absolute difference is modest (approximately 2 mm), this reduced separation may increase the risk of PA or ATA injury during tibial exposure, retraction, and bone cuts in TKA, especially in female patients [37]. This underscores the value of review of pre-existing ipsilateral knee MRI, selective preoperative imaging, and intraoperative vigilance. This review examined the prevalence of PA variants and their role in vascular injury during primary and revision TKA.

## Material and methods

### Literature search and screening

This scoping review followed Preferred Reporting Items for Systematic Reviews and Meta-Analyses extension for Scoping Reviews (PRISMA-ScR) guidelines and was registered in International Prospective Register of Systematic Reviews (PROSPERO) (CRD42024560429). A comprehensive literature search was conducted in EMBASE, MEDLINE, and Scopus in February 2025 using keywords related to PA anatomy and vascular complications: (“aberrant popliteal artery” OR “aberrant anterior tibial artery” OR “popliteal artery variation” OR “popliteal artery anomalies” OR “popliteal artery anatomy”) AND (“intraoperative vascular injury” OR “vascular complications” OR “surgical complications”).

Two reviewers independently screened titles and abstracts for relevance. Discrepancies were resolved through discussion and consensus, with senior author adjudication when required. Screening was performed using Covidence software. Titles and abstracts were reviewed, followed by full-text screening. Eligible studies (2019–2025) included those examining PA anatomical variation, prevalence, or imaging in the context of TKA. Case reports describing intraoperative or postoperative vascular complications involving aberrant PA anatomy during TKA were also included (Table 1). Studies describing unicompartmental or revision TKA were included only if they provided relevant information on PA anatomy or vascular injury risk applicable to TKA. The 5-year window ensured alignment with current surgical practices.

**Table 1:** Studies included in Preferred Reporting Items for Systematic Reviews and Meta-Analyses systematic review.

Study (year)	Article type	Article description
Jang et al. (2018)	Case report	Preoperative MRI detection of aberrant ATA in TKA
Wanken et al. (2019)	Case report	Pseudoaneurysm after TKA due to high takeoff of the posterior tibial artery
Yazici (2023)	Case report	Intraoperative identification of aberrant artery during TKA
Gosslau et al. (2022)	Cohort study	Cohort including patients with PA complications during knee surgeries
Runge et al. (2020)	Case report	Vascular injury in TKA due to variant PA
Marin-Concha et al. (2021)	Imaging-based study	Systematic review of PA anatomy variations and prevalence
Schuster et al. (2024)	Imaging-based study	MRI-based classification of surgical risk zones for PA injury
Kimura et al. (2022)	Imaging-based study	Relationship between PA variants and tibial cortex proximity
Hamahashi et al. (2022)	Imaging-based study	MRI-based assessment of gender differences in PA anatomy
Olewnik et al. (2019)	Cadaver-based study	Morphometric analysis of PA branching patterns
Aragonés et al. (2020)	Cadaver-based study	Classification of PA variants and surgical implications

Exclusion criteria were animal studies, abstracts, technique papers, opinion pieces, reviews, book chapters, non-English articles, and studies not involving the PA. Per scoping review methodology, no formal quality assessment of included studies was performed.

This study was exempt from institutional review board approval.

### Data extraction

Key information extracted included article title, author, study design, publication year, level of evidence, number of knees analyzed, patient demographics, and radiographic findings. For anatomy-focused studies, anatomical variants and their definitions were recorded. For complication-related studies, relevant surgical outcomes were documented. All data were organized using Microsoft Excel (Microsoft Office 2016).

## Results

The initial search yielded 6164 articles. After removing 1070 duplicates, 5093 titles and abstracts were screened. Thirty-eight full-text articles were reviewed, and 11 met inclusion criteria to identify high-risk anatomical variants.

### Study characteristics

Four studies reported TKA-related arterial injuries in patients with aberrant PA anatomy (Table 2) [[2], [3], [4],^16^]. One additional study described a PA injury without detailing the arterial anatomy (Table 3) [5]. Another provided prevalence data from a single center (Table 4) [14]. Five studies used imaging or cadaveric methods to assess anatomical variation or define surgical risk zones (Tables 5 and 6) [15,[18], [19], [20],^37^].

**Table 2:** Case reports with aberrant anatomy of popliteal artery identified.

Author and year	Patient characteristics	Preoperative imaging	Knee surgery	Aberrant popliteal artery anatomy	Outcome
Jang et al. (2018)	61-year-old woman with right knee osteoarthritis; no prior knee surgery	MRI detected aberrant ATA	Right TKA	PA bifurcated at the knee joint level; ATA coursed anteriorly to the tibioperoneal trunk	Successful TKA without complications
Wanken et al. (2019)	76-year-old woman presented to emergency department 2 weeks after elective right TKA (performed w/o tourniquet)	Not specified	Right TKA	Aberrant PT artery with a high division	Coil embolization successfully treated a pseudoaneurysm
Yazici (2023)	77-year-old woman with bilateral knee osteoarthritis undergoing left TKA	Not specified	Left TKA	4 mm arterial opening observed anterior to the joint capsule at the joint line during implant placement	Bleeding vessel clamped but not repaired; no postoperative complications
Gosslau et al. (2022)	16 cases of PA injury following knee surgery	Not specified	11 TKA, 2 revision TKAs	High branching of ATA and PT artery (n = 2); injuries specifically involved these variants	All limbs preserved; 6 cases with functional impairment

**Table 3:** Case reports with popliteal artery anatomy not identified.

Study	Patient characteristics	Preoperative imaging	Knee surgery	Outcome
Runge et al. (2020)	38-year-old man with post-traumatic osteoarthritis; BMI 37.5; nonsmoker; no significant medical history	X-ray: retained hardware, severe medial joint space narrowing, osteophytes, subchondral sclerosis	Right TKA	Developed anterior shin pain and lower leg swelling 2 h postop; thrombus in distal PA. Open thrombectomy removed 2 medium and 1 small clot.

**Table 4:** Imaging studies on popliteal artery anatomy: prevalence and surgical implications.

Study	Sample size	Imaging modality	Aberrant popliteal artery prevalence	Other key finding(s)
Marin-Concha et al. (2021)	280 knees	MRI	2.9%	Proton density fat saturated MRI best for detection
Schuster et al. (2024)	1589 knees	MRI + long leg radiographs	2.1%	Identified 3 risk zones for surgery
Kimura et al. (2022)	129 patients	MRI	4.7% (proximal branching)	Distance from PA to tibial cortex significantly shorter in proximal branching
Hamahashi et al. (2022)	105 patients	MRI	11.4% (proximal ATA branching)	Risk of PA injury higher in women

**Table 5:** Popliteal artery and anterior tibial artery measurements: surgical implications.

Study	Measurement	Mean ± SD (mm)	Key findings
Schuster et al. (2024)	Anteroposterior distance of ATA to tibial head	6.6 ± 2.5	ATA is close to tibial structures, varying by surgical risk zones
	ATA distance to PCL footprint	2.7 ± 1.6	Risk of injury during PCL reconstruction
	ATA distance under fibular head	1.0 ± 0.6	Close proximity increases risk in high tibial osteotomy
Kimura et al. (2022)	PA/ATA distance to tibial cortex (proximal branching)	1.8 ± 1.1	Significantly shorter in proximal branching variant
	PA/ATA distance to tibial cortex (distal branching)	6.1 ± 2.6	Safer distance in distal branching
Hamahashi et al. (2022)	PA to tibial cortex at zone A (5–10 mm distal to tibial plateau)	5.5 ± 1.9	PA gradually shifts laterally with distal progression
	PA to tibial cortex at zone D (35–40 mm distal)	12.5 ± 2.3	Distance smaller in women, higher risk of PA injury

**Table 6:** Popliteal artery branching patterns identified in cadaveric studies.

Study	Sample size	Popliteal artery branching patterns	Prevalence of each	Other key findings
Olewnik et al. (2019)	100 lower limbs	Type I: PA divides into ATA and common junction for PTA and peroneal arteries (subgroup variations)	72%	Focused on morphometric measurements, common patterns involve ATA, PTA, and peroneal arteries
		Type II: PA divides into ATA and PTA	8%	
		Type III: trifurcation	12%	
		Type IV: PTA divides into ATA and peroneal artery Aplasia of PTA	8%	
Aragonés et al. (2020)	260 lower limbs	Pattern 1: PA divides into ATA and PTA distal to the popliteal muscle	94.7%	Further detailed classification with variations in the PEA origin and division levels
		Pattern 2.a: PA divides into ATA and PTA, PEA arises from PTA, DPTA continues	1.6%	
		Pattern 2.b: PA divides into ATA and PTA, PEA from ATA	0.8%	
		Pattern 3: trifurcation (ATA, PTA, PEA)	2%	

DPTA, distal posterior tibial artery; PEA, peroneal artery.

Among the 4 case reports, the mean patient age was 63.0 ± 18.2 years; all had osteoarthritis, and 3 of the 4 (75%) were women [2,3,5,16]. One patient had a body mass index >30, with no other major comorbidities noted [5].

Gosslau et al. reported 16 vascular injuries during knee surgery: 11 during primary TKA, 2 during revision TKA, 1 during arthroscopy, and 2 during high tibial osteotomy [4]. The average age was 67.5 years, and 37.5% (6/16) were women [4]. Two patients had high branching of both the anterior and PT arteries, which likely contributed to the vascular injury [4].

### Imaging-based studies to identify anatomical variations of PA relevant to TKA

Anatomical variation of the ATA and its clinical implications during TKA have been well described using MRI [14,15,18,37]. Reported prevalence of aberrant ATA ranges from 0.4% to 6% [14]. Marin-Concha et al. found a 3.2% prevalence in 280 knees, while Schuster et al. reported 2.1% in a larger cohort of 1589 knees [14,15]. Schuster et al. additionally reported that in 2 cases where bilateral imaging was available, the ATA variant was present on only 1 side, suggesting that contralateral asymmetry of PA branching patterns can occur [15].

The aberrant ATA’s close proximity to key surgical landmarks, such as the posterior capsule and posterior cruciate insertion site where the transverse tibial cut is usually made during TKA, was described by Schuster et al., measuring 6.6 ± 2.5 mm to the most posterior part of the posterior cruciate ligament footprint at the level of the tibial plateau, 2.7 ± 1.6 mm from the posterior cruciate ligament footprint itself, and 1.0 ± 0.6 mm below the fibular apex [15]. These findings underscore the vulnerability of these zones in high-division variants during TKA.

The ATA and PA position relative to the posterior proximal tibial cortex during knee extension was assessed by Kimura et al [18]. In cases with proximal branching (4.7% prevalence), the distance between the PT cortex and the vessel was significantly shorter (1.8 ± 1.1 mm) than in distal branching (6.1 ± 2.6 mm, *P* < .05), increasing injury risk [18].

Hamahashi et al. used preoperative MRI in flexed knees and found high ATA branching (40 mm within the joint line) in 11.4% of cases, predominantly among women [37]. Although often distal to the tibial cut, altered ATA courses and close PA positioning may heighten risk during deep retraction or posterior exposure. One case of ATA bifurcation at the joint line emphasized the importance of preoperative imaging to identify vascular variants [17].

### Cadaveric studies to identify anatomical variations of the PA relevant to TKA

An investigation of the anatomical variations in the branching patterns of the PA, specifically focusing on the ATA, posterior tibial artery (PTA), and peroneal artery was performed by Olewnik et al [19]. The study involved dissection of 100 lower limbs fixed in formalin, with morphometric measurements taken to analyze the course and morphology of the PA's terminal branches [19]. The most common branching pattern (72%) was type I, in which the PA divides into the ATA and a tibioperoneal trunk, which then bifurcates into the PT artery and peroneal artery [19]. Type I was further subdivided into 2 subgroups based on whether the ATA or the common junction had the larger diameter [19]. Other variations included type II (8%), where the PA divided into the ATA and PT artery type III (12%) involving trifurcation into the ATA, PTA, and peroneal artery, type IV (8%) where the PTA divided into the ATA and peroneal artery, and the absence of the PTA (8%) in some specimens [19].

These atypical configurations, particularly high trifurcations and absent or anomalous branches, may result in arteries coursing more anteriorly or closer to the PT cortex. As a result, they are more susceptible to iatrogenic injury. Additionally, rare patterns such as PTA absence reduce the redundancy of the lower limb’s vascular supply, heightening the clinical consequences of intraoperative vessel damage.

The anatomical variability of PA branching patterns was examined using cadaveric dissection, and multivariate analysis of 260 lower limbs was investigated by Aragonés et al [20]. The authors proposed a simplified classification of PA branching into 3 main patterns: pattern 1 (94.7%), a typical bifurcation of the PA into the anterior and PT arteries at or distal to the lower border of the popliteal muscle; pattern 2 (3.3%), a high bifurcation occurring proximal to this level; and pattern 3 (2%), a trifurcation into the anterior tibial, PT, and peroneal arteries [20]. These represent variations in branching level and morphology, rather than positional anomalies, and were not associated with differences based on sex or laterality.

While the cadaveric studies identified several branching variations of the PA, Kim et al. further characterized type II variations as forms of “high division” of the PA, where any of the 3 distal branches arise at or above the knee joint [[17], [18], [19], [20]]. These high divisions result in more proximal branching, which, as Kimura et al. noted, can significantly reduce the distance between the artery and PT cortex, increasing the risk of arterial injury during TKA procedures [18].

## Discussion

### Importance of preoperative detection of aberrant PA in TKA

This review underscored the importance of recognizing PA anatomical variations before TKA. While most patients lack vascular anomalies, high-branching variants significantly increase the risk of intraoperative injury, as shown by several authors [[2], [3], [4],^16^]. Preoperative detection enables better surgical planning and may prevent serious complications such as hemorrhage, emergent bypass, ischemia, pseudoaneurysm, or amputation. The challenge remains identifying these variants on imaging, as MRI is not routinely included in the preoperative workup for arthroplasty patients [21].

Given the variability in PA anatomy, surgeons must account for these differences during preoperative planning. Jang et al. used MRI to identify a high bifurcation of the PA with an anteriorly coursing ATA in a TKA patient who presented with rapidly worsening pain after a traumatic fall [16]. Recognition of this anomaly allowed the team to avoid penetrating the posterior cortex and to use an osteotome for the tibial cut [16]. Precise mapping of vascular anatomy via MRI can be critical for preventing intraoperative injury and optimizing outcomes.

Jang et al. reported a successful TKA after preoperative identification of an aberrant ATA, while other studies without recognition of vascular anomalies described serious complications, including pseudoaneurysm and limb ischemia [2,3,16]. Amputation has occurred in up to 42% of TKA cases involving PA injury [31,38,39]. These outcomes highlight the importance of preoperative imaging in surgical planning. Though vascular studies like MRI are not routinely used due to cost and low yield, targeted imaging may be justified in high-risk patients, such as those with peripheral arterial disease, malignancy, or unexplained weight loss [6,9,[26], [27], [28], [29]].

### Implication of high division pattern of popliteal artery on TKA

High-division variants of the PA, where bifurcation occurs proximal to the joint line, can increase the risk of vascular injury during TKA due to the closer proximity of arterial branches to the PT cortex and posterior knee capsule. This high-division pattern occurs in 3.3% of specimens and involves variable branching combinations was shown by Aragonés et al [19].

A variant where the ATA courses anterior to the tibioperoneal trunk, in contact with the PT cortex (type IIA variant, per Kim et al.’s modified Lippert classification), was described by Jang et al [16]. This variation, present in up to 6% of the population, was identifiable on preoperative MRI, allowing for surgical planning and risk mitigation [16,17].

Vascular injuries during knee surgery are often associated with aberrant arterial anatomy, particularly high bifurcations of the PA. High-origin ATA or peroneal arteries have been implicated in direct surgical trauma. Gosslau et al. identified 2 such cases among 16 vascular injuries during knee procedures [4]. Yazici et al. reported a peroneal artery transection due to a proximally dividing PA near the joint line [3]. Wanken et al. described injury to a high takeoff ATA during TKA, and Runge et al. noted popliteal vein damage with presumed PA torsion in a patient with aberrant branching [2,5]. These cases highlight how high-division variants can significantly narrow the posterior safe zone and may go undetected without preoperative imaging [[2], [3], [4], [5]].

Together, these studies underscored the surgical relevance of high-division PA anatomy. By reducing the safe operative corridor, these variants increase susceptibility to arterial injury. Preoperative imaging, particularly MRI or vascular ultrasound, can help identify at-risk patients and guide intraoperative strategy, including avoiding the use of tourniquet, careful saw cuts, exposing the posterior cortex and making the posterior cuts under direct visualization, careful placement of posterior retractors, and avoiding excessive posterior releases.

### Prevalence of aberrant PA and patterns of preoperative imaging in TKA

The true prevalence of aberrant PA anatomy in TKA patients is likely underrecognized, in part due to inconsistent imaging practices and potential medicolegal factors [1,3,7,14,15,[18], [19], [20],^22^,^35^,^37^]. Reported prevalence of abnormal arterial branching ranges from 0.4% to 6%, reflecting variability in detection and reporting [14]. TKA planning typically relies on standard radiographs, which cannot detect vascular anomalies [21,22]. Advanced imaging like CT angiography is rarely used unless prompted by trauma or atypical presentation. However, preoperative MRI, often obtained for other reasons, may offer valuable vascular detail [21,22,40,41]. While routine MRI is debated due to cost concerns, lack of vascular imaging may leave high-risk variants undetected, increasing the risk of intraoperative injury [22,40,41].

In a study evaluating the frequency of MRI in the year prior to TKA, Rudisill et al. found that 7.68% of 731,066 TKA patients received an MRI within 1 year preoperatively, and 51.9% of 731,066 within 3 months [22]. Predictors of MRI use included younger age, female sex, higher Elixhauser Comorbidity Index, and Medicaid or commercial insurance [22].

Furthermore, documentation of arterial anatomy is often absent even in cases with postoperative complications. [5] This lack of imaging combined with possible medicolegal pressures surrounding intraoperative complications may contribute to both clinical underrecognition and academic underreporting of PA injury during TKA.

### Study limitations

This review was limited by the scarcity of high-quality literature, preventing more in-depth quantitative analyses and restricting study quality assessment. Most available studies were retrospective, case reports, or small series, introducing potential bias. Despite these limitations, this work synthesized current evidence on PA variations and highlights the need for larger, well-designed studies to better define their prevalence and surgical implications during TKA.

### Future research to prevent vascular injury post-TKA

Future research should focus on refining surgical techniques to minimize the risk of PA injury or surgical adaptations when aberrant vascular anatomy is detected [42]. Awareness of aberrant PA anatomy during preoperative planning allows the surgeon to modify the surgical approach accordingly [16]. Intraoperatively, a subperiosteal posterior capsular release and carefully positioned posterior retractors with the knee in flexion facilitate a safer tibial cut [13,43,44]. For patients with high-risk vascular anatomy, performing the procedure in an inpatient hospital setting with vascular surgery support immediately available provides an added margin of safety [3,4,45]. When an aberrant PA is identified, additional precautions include avoiding tourniquet use, performing controlled saw cuts of the posterior femoral condyles and proximal tibia with the knee flexed, minimizing osteotome use when excising posterior condylar osteophytes, maintaining retractors in direct contact with the posterior cortex, and limiting excessive posterior capsular release [9,26,27,29,[45], [46], [47], [48], [49]]. Some robotic-assisted TKA systems incorporate haptic boundaries and real-time feedback mechanisms that may help prevent sawblade excursion beyond the intended bone resection zone, potentially reducing the risk of iatrogenic vascular injury [50]. This underscores the importance of understanding how we can detect aberrant PA anatomy and prevent TKA vascular complications.

## Conclusions

PA anatomical variations in the TKA literature may be more common than previously reported, partly due to limited use of advanced imaging in preoperative TKA assessment. Cadaveric and imaging studies have identified high-risk variants, such as high bifurcation and trifurcation patterns, that increase the risk of vascular injury. Identifying these variants preoperatively may improve surgical planning and reduce complications. Vascular proximity to key landmarks may also vary by gender, putting women at higher risk. Routine imaging is not warranted, but review of pre-existing ipsilateral MRI, selective imaging in high-risk patients may prevent catastrophic complications and inform intraoperative strategy.

## CRediT authorship contribution statement

**Hiren Parekh:** Writing – review & editing, Writing – original draft, Methodology, Investigation, Data curation, Conceptualization. **Bohdanna Zazulak:** Writing – review & editing, Supervision, Methodology, Investigation, Conceptualization. **Adam V. Daniel:** Writing – review & editing, Methodology, Investigation, Conceptualization. **Kinjal Vasavada:** Writing – review & editing, Visualization. **Elie Mansour:** Writing – review & editing, Investigation. **Michael Medvecky:** Writing – review & editing, Supervision, Project administration.

## Conflict of interest

B. Zazulak: Book royalties for “Master Your Core”, TCK Publishing and Integrative Sports Injury Reduction and Rehabilitation: A Frontier of Lifestyle Medicine—paid presentation Lifestyle Medicine Conference/NJ 10/23; all other authors declare no potential conflicts of interest.

For full disclosure statements refer to https://doi.org/10.1016/j.artd.2026.101975.

## Appendix A Supplementary Data

Conflict of Interest Statement for Parekh

Conflict of Interest Statement for Zazulak

Conflict of Interest Statement for Daniel

Conflict of Interest Statement for Vasavada

Conflict of Interest Statement for Mansour

Conflict of Interest Statement for Medvecky
