# Supplementary material for: Popliteal Artery Injury Risk in Total Knee Arthroplasty Related to Anatomic Variations: A Scoping Review
Source: Arthroplast Today. 2026 Feb 25;38:101975. doi: 10.1016/j.artd.2026.101975 (PMC12954335; doi:10.1016/j.artd.2026.101975)
Supplement: Conflict of Interest Statement for Daniel [file mmc3.docx]

# INDIVIDUAL CONFLICT OF INTEREST STATEMENT

***American Association of Hip and Knee Surgeons***

(Adopted from the American Academy of Orthopaedic Surgeons disclosure statement)

The following form **must be filled out completely and submitted by each author (example, 6 authors, 6 forms).**

**All items require a response. If there is no relevant disclosure for a given item, enter "*None*.”**

**Popliteal Artery Variations and Vascular Injury Risk in Total Knee Arthroplasty: A Scoping Review**

1. Royalties from a company or supplier (The following conflicts were disclosed)

N/A

2. Speakers bureau/paid presentations for a company or supplier (The following conflicts were disclosed)

N/A

3A. Paid employee for a company or supplier (The following conflicts were disclosed)

N/A

3B. Paid consultant for a company or supplier (The following conflicts were disclosed)

N/A

3C. Unpaid consultants for a company or supplier (The following conflicts were disclosed)

N/A

4. Stock or stock options in a company or supplier (The following conflicts were disclosed)

N/A

5. Research support from a company or supplier as a Principal Investigator (The following conflicts were disclosed)

N/A

6. Other financial or material support from a company or supplier (The following conflicts were disclosed)

N/A

7. Royalties, financial or material support from publishers (The following conflicts were disclosed)

N/A

8. Medical/Orthopaedic publications editorial/governing board (The following conflicts were disclosed)

N/A

9. Board member/committee appointments for a society (The following conflicts were disclosed)

N/A

**Each author must sign AND print or type his/her name, date and submit a separate form**

In addition, one BLINDED Conflict of Interest form (no author names used) should be submitted per manuscript with all author disclosures.


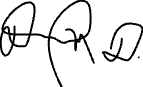


Adam V. Daniel, MD 08/13/2025 1921


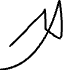


Author Name (Print or Type) Author Signature Date
