# Supplementary material for: Popliteal Artery Injury Risk in Total Knee Arthroplasty Related to Anatomic Variations: A Scoping Review
Source: Arthroplast Today. 2026 Feb 25;38:101975. doi: 10.1016/j.artd.2026.101975 (PMC12954335; doi:10.1016/j.artd.2026.101975)
Supplement: Conflict of Interest Statement for Vasavada [file mmc4.pdf]

INDIVIDUAL CONFLICT OF INTEREST STATEMENT

American Association of Hip and Knee Surgeons

(Adopted from the American Academy of Orthopaedic Surgeons disclosure statement)

The following form **must be filled out completely and submitted by each author (example, 6 authors, 6 forms).**
All items require a response. If there is no relevant disclosure for a given item, enter "*None*."

Popliteal Artery Variations and Vascular Injury Risk in Total Knee Arthroplasty: A Scoping Review

1. Royalties from a company or supplier (The following conflicts were disclosed)
None
2. Speakers bureau/paid presentations for a company or supplier (The following conflicts were disclosed)
None

3A. Paid employee for a company or supplier (The following conflicts were disclosed)
None

3B. Paid consultant for a company or supplier (The following conflicts were disclosed)
None

3C. Unpaid consultants for a company or supplier (The following conflicts were disclosed)
None

4. Stock or stock options in a company or supplier (The following conflicts were disclosed)
None
5. Research support from a company or supplier as a Principal Investigator (The following conflicts were disclosed)
None
6. Other financial or material support from a company or supplier (The following conflicts were disclosed)
None
7. Royalties, financial or material support from publishers (The following conflicts were disclosed)
None
8. Medical/Orthopaedic publications editorial/governing board (The following conflicts were disclosed)
None
9. Board member/committee appointments for a society (The following conflicts were disclosed)
None

Each author must sign AND print or type his/her name, date and submit a separate form

In addition, one BLINDED Conflict of Interest form (no author names used) should be submitted per manuscript with all author disclosures.

Kinjal Vasavada	[signature]	8/14/25
Author Name (Print or Type)	Author Signature	Date
